# Prison Buprenorphine Implementation and Postrelease Opioid Use Disorder Outcomes

**DOI:** 10.1001/jamanetworkopen.2024.2732

**Published:** 2024-03-18

**Authors:** Benjamin J. Bovell-Ammon, Shapei Yan, Devon Dunn, Elizabeth A. Evans, Peter D. Friedmann, Alexander Y. Walley, Marc R. LaRochelle

**Affiliations:** 1 Departments of Medicine and of Healthcare Delivery and Population Sciences, Baystate Health, Springfield, Massachusetts; 2 Department of Medicine, Boston Medical Center and Boston University Chobanian & Avedisian School of Medicine, Boston, Massachusetts; 3 Massachusetts Department of Public Health, Boston, Massachusetts; 4 Department of Health Promotion and Policy, University of Massachusetts Amherst School of Public Health & Health Sciences, Amherst; 5 Office of Research and Department of Medicine, University of Massachusetts Chan Medical School—Baystate and Baystate Health, Springfield

## Abstract

**Importance:**

Agonist medications for opioid use disorder (MOUD), buprenorphine and methadone, in carceral settings might reduce the risk of postrelease opioid overdose but are uncommonly offered. In April 2019, the Massachusetts Department of Correction (MADOC), the state prison system, provided buprenorphine for incarcerated individuals in addition to previously offered injectable naltrexone.

**Objective:**

To evaluate postrelease outcomes after buprenorphine implementation.

**Design, Setting, and Participants:**

This cohort study with interrupted time-series analysis used linked data across multiple statewide data sets in the Massachusetts Public Health Data Warehouse stratified by sex due to differences in carceral systems. Eligible participants were individuals sentenced and released from a MADOC facility to the community. The study period for the male sample was January 2014 to November 2020; for the female sample, January 2015 to October 2019. Data were analyzed between February 2022 and January 2024.

**Exposure:**

April 2019 implementation of buprenorphine during incarceration.

**Main Outcomes and Measures:**

Receipt of MOUD within 4 weeks after release, opioid overdose, and all-cause mortality within 8 weeks after release, each measured as a percentage of monthly releases who experienced the outcome. Segmented linear regression analyzed changes in outcome rates after implementation.

**Results:**

A total of 15 225 individuals were included. In the male sample there were 14 582 releases among 12 688 individuals (mean [SD] age, 35.0 [10.8] years; 133 Asian and Pacific Islander [0.9%], 4079 Black [28.0%], 4208 Hispanic [28.9%], 6117 White [41.9%]), a rate of 175.7 releases per month; the female sample included 3269 releases among 2537 individuals (mean [SD] age, 34.9 [9.8] years; 328 Black [10.0%], 225 Hispanic [6.9%], 2545 White [77.9%]), a rate of 56.4 releases per month. Among male participants at 20 months postimplementation, the monthly rate of postrelease buprenorphine receipt was higher than would have been expected under baseline trends (21.2% vs 10.6% of monthly releases; 18.6 additional releases per month). Naltrexone receipt was lower than expected (1.0% vs 6.0%; 8.8 fewer releases per month). Monthly rates of methadone receipt (1.4%) and opioid overdose (1.8%) were not significantly different than expected. All-cause mortality was lower than expected (1.9% vs 2.8%; 1.5 fewer deaths per month). Among female participants at 7 months postimplementation, buprenorphine receipt was higher than expected (31.6% vs 9.5%; 12.4 additional releases per month). Naltrexone receipt was lower than expected (3.4% vs 7.2%) but not statistically significantly different. Monthly rates of methadone receipt (1.1%), opioid overdose (4.8%), and all-cause mortality (1.6%) were not significantly different than expected.

**Conclusions and Relevance:**

In this cohort study of state prison releases, postrelease buprenorphine receipt increased and naltrexone receipt decreased after buprenorphine became available during incarceration.

## Key Points

**Question:**

Did trends in postrelease treatment and outcomes for opioid use disorder change after Massachusetts prison system implemented buprenorphine treatment during incarceration?

**Findings:**

In this cohort study of 15 225 adults incarcerated and released from prisons in Massachusetts, when buprenorphine treatment began to be offered during incarceration (previously only naltrexone was available), postrelease buprenorphine receipt in the community increased, accompanied by a smaller decrease in naltrexone receipt. When stratified by sex, there was also a small decrease in postrelease all-cause mortality in the male cohort (and a decrease in postrelease opioid overdose in the female cohort that was not statistically significant).

**Meaning:**

These results suggest that offering buprenorphine during incarceration might improve treatment receipt during the critical postrelease period.

## Teaser

This cohort study of adults who were incarcerated and released from prisons in Massachusetts examines the association of buprenorphine treatment with usage rates and health outcomes.

## Introduction

Despite a high risk of opioid overdose in the first weeks after release from incarceration,^1,2,3,4,5,6^ most jails and prisons in the US do not routinely offer medications for opioid use disorder (MOUD) to patients during incarceration or upon release.^7,8,9,10,11^ The National Academy of Sciences, Engineering, and Medicine recommends that all forms of MOUD approved by the Food and Drug Administration (FDA) be available and accessible in all care venues, particularly carceral settings,^12^ and more jurisdictions have been adopting MOUD in recent years.^13^

All 3 forms of MOUD—buprenorphine, methadone, and naltrexone—effectively promote recovery and prevent complications of substance use,^14,15,16,17^ including among incarcerated and recently released individuals.^18,19,20,21,22,23,24^ In particular, the agonist medications buprenorphine and methadone reduce opioid overdoses.^14,16,17,22^ Randomized trials and observational studies have shown that MOUD treatment during incarceration (as opposed to referral to initiate MOUD in the community postrelease) is the best way to ensure effective protection during the initial reentry period when the risk of opioid overdose is highest.^19,25,26,27,28,29,30^ However, few real-world, population-level studies have examined how implementation of agonist MOUD in carceral settings affects postrelease treatment engagement and outcomes.

Massachusetts has been heavily impacted by the opioid overdose epidemic in the US.^31,32,33,34^ Until recently, access to MOUD in the state prison system, the Massachusetts Department of Correction (MADOC), was restricted to a few pregnant women who received methadone and participants (both male and female) in the Medication Assisted Treatment Re-Entry Initiative (MATRI), which operated throughout MADOC since 2014 and offered a prerelease dose of long-acting injectable naltrexone (the opioid antagonist medication) and linkage to ongoing treatment after release. However, a new state law (Chapter 208 of the Massachusetts Acts of 2018^35^) mandated that MADOC offer all 3 FDA-approved MOUDs to eligible patients at a minimum of 4 pilot facilities starting in April 2019. Pursuant to this law, MADOC implemented sublingual buprenorphine treatment in its 2 prisons with female populations and 2 of its 11 prisons for male populations in April 2019; another facility for males was added in January 2020 (together, these 3 prisons for males accounted for about 28% of male releases at that time). (Due to greater logistical and regulatory requirements, methadone was not added until December 2020.) As part of this MOUD program, universal OUD screening was implemented. Initially the program focused on continuing buprenorphine for individuals who had been prescribed it prior to incarceration, and gradually they increased capacity to initiate buprenorphine treatment. For individuals receiving buprenorphine during incarceration, clinicians arranged appointments for postrelease care in the community and provided a bridge script upon release. Our study considered the MADOC policy change in April 2019 as a natural experiment—an opportunity to study the population-level association of carceral buprenorphine implementation and postrelease MOUD treatment and outcomes. We hypothesized that after April 2019 postrelease buprenorphine receipt would increase, postrelease naltrexone and methadone receipt would not change, and postrelease opioid overdoses and all-cause deaths would decrease.

## Methods

### Data Source and Sample Selection

We used an interrupted time series design to study the association of the implementation of buprenorphine in five MADOC prisons with postrelease MOUD and outcomes among recent releases. We used 2014 to 2020 data from the Massachusetts Public Health Data Warehouse, a statewide data platform created by the Massachusetts Department of Public Health, which individually links the state’s All-Payer Claims Database (APCD) with data from multiple other data sources, including MADOC, the Prescription Monitoring Program (PMP), the Bureau of Substance Addiction Services (BSAS) funded treatment programs, the Acute Care Hospital Case Mix file (discharge records), the Massachusetts Ambulance Trip Record Information System (MATRIS), and the Registry of Vital Records and Statistics death certificate data. Detailed information about the data sets and linkage process has been previously published^33,36^ and is available online.^37^ The Boston University Medical Campus institutional review board determined that research with the Massachusetts Public Health Data Warehouse is not human participant research. Reporting in this study followed the REporting of studies Conducted using Observational Routinely-collected health Data (RECORD) guidelines,^38^ an extension of the Strengthening the Reporting of Observational Studies in Epidemiology (STROBE) reporting guidelines.

We used data on all releases to the community from the sentenced MADOC population. Because of structural differences in the carceral systems for males and females in Massachusetts, we stratified all analyses by sex. Because our study relied on MADOC demographic data, we used the term *sex* with the binary categories of male and female, which were the only 2 categories provided by this data source. Generally, individuals in Massachusetts detained while awaiting court proceedings (pretrial) and those serving shorter sentences (less than 2.5 years) for criminal convictions remain in the custody of a county house of correction (ie, jail). Convicted individuals (both male and female) who receive sentences greater than 2.5 years are transferred to the state prison system, MADOC. Many counties do not have facilities for female populations and contract with another jurisdiction. Historically, most counties contracted with the state to house females for them in MADOC’s facilities for females. However, 4 counties in central and western Massachusetts ended their contract with MADOC in late 2014 and started sending females to a regional female correctional facility run by neighboring Hampden County.^39^ Similarly, 3 eastern Massachusetts counties moved their incarcerated women from MADOC to the Suffolk County House of Correction in November 2019.^40^ To avoid discontinuities in the makeup of our female sample, we limited the study period to January 2015 to October 2019, during which time the MADOC held all longer-sentenced individuals as well as shorter-sentenced individuals from 5 counties (Dukes, Essex, Middlesex, Norfolk, and Plymouth)—representing approximately 55% of the entire sentenced female population in the state.^41^ Meanwhile, we included all available months of data for the male sample, January 2014 to November 2020, as we did not have to account for discontinuities during this time (with December releases excluded because we could not measure their postrelease outcomes [described later]).

### Measures

Our 3 primary outcomes were the receipt of buprenorphine, naltrexone, and methadone, respectively, within 4 weeks (28 days) after release from MADOC. We identified buprenorphine receipt as any prescription fill of buprenorphine in the PMP, any pharmacy claim, or any Healthcare Common Procedure Coding System (HCPCS) or *International Classification of Diseases, Tenth Revision, Procedure Coding System* (*ICD-10-PCS*) code for administration in the APCD, including all sublingual, injectable, and implantable formulations approved for OUD treatment. We identified naltrexone receipt as any pharmacy claim or any HCPCS or *ICD-10-PCS* code for administration in the APCD of the injectable extended-release formulation. We identified methadone receipt as any HCPCS or *ICD-10-PCS* procedure code for methadone administration in the APCD or enrollment in a methadone treatment program in the BSAS data. We provide details of the data sets and definitions used to identify each type of MOUD in eTable 1 in Supplement 1.

Our 2 secondary outcomes were opioid-related overdose (fatal and nonfatal combined) and death (from any cause), respectively, within 8 weeks (56 days) after release from MADOC. Deaths were identified using death certificates. Opioid overdoses were identified in 3 possible ways: first, an emergency department or hospital encounter for an acute opioid overdose based on *International Classification of Diseases, Ninth Revision (ICD-9)* or *International Statistical Classification of Diseases, Tenth Revision, Clinical Modification (ICD-10-CM)* diagnosis codes in the Acute Care Hospital Case Mix file; second, an ambulance encounter for an acute opioid overdose in the Massachusetts Ambulance Trip Record Information System; and, third, an opioid-related death based on *ICD-10* codes for the underlying and contributing causes of death on a death certificate. We provide details of the data sets and definitions used to identify opioid overdoses and all-cause mortality in eTable 2 in Supplement 2.

We also used descriptive information about released individuals from the MADOC data set: sex, age at release, race and Hispanic ethnicity, length of incarceration, governing offense (property, drug, sex, other offense against the person, or other), presence of serious mental illness, MATRI program participation, and parole status upon release. The following race and ethnicity classifications were provided by MADOC from their release records database: Hispanic (of any race); non-Hispanic American Indian; non-Hispanic Asian or Pacific Islander; non-Hispanic Black; non-Hispanic White; and other race (combined with non-Hispanic American Indian in the MADOC data set). We note that, because the MADOC data set only accounted for time in MADOC specifically, the length of incarceration variable might be artificially low in some cases—primarily for individuals transferred to MADOC from another jurisdiction such as a county jail. Additionally, at the start of our study period, MADOC’s definition of serious mental illness was limited primarily to psychotic and bipolar disorders, but then the definition was broadened starting in 2019 to also include major depressive disorders, anxiety disorders, posttraumatic stress disorder, and others.

### Statistical Analysis

Our unit of analysis was releases (ie, at the event level, so the same person could have multiple releases in our sample). We aggregated data by month and calculated each outcome rate as the percentage of releases in each month who experienced the outcome. Segmented linear regression tested for changes in each outcome after MADOC’s new MOUD program started in April 2019. For each outcome, we first considered a saturated model with 4 terms: baseline level (intercept), baseline trend, postintervention change in level, and postintervention change in trend. As needed, a manual stepwise backward elimination approach removed terms with *P* > .20 from the model to generate a more parsimonious model.^42^ Each model also used stepwise autoregression to automatically test and adjust for temporal autocorrelation, with an initial order of 13 months. Using the final regression model results for each outcome, a multivariate delta method estimated the cumulative difference at the end of follow-up (ie, absolute percentage-point difference in the monthly rate) comparing the actual modeled outcome value with a counterfactual outcome value that would have been expected if baseline trends had continued (ie, if the intervention had not occurred).^43^ For units that are easier to interpret, we multiplied this estimate by the average number of monthly releases (across the study period) to convert the percentage-point rate difference to a difference in the number of releases per month. Because the male sample’s follow-up period included the beginning of the COVID-19 pandemic, we conducted a sensitivity analysis by truncating follow-up with male participants after December 2019. We used SAS version 9.4 and SAS Studio version 3.81 for analyses (SAS Institute Inc). Hypothesis tests were 2-sided with *P* ≤ .05 as significant. Data analysis occurred from February 14, 2022, to January 16, 2024.

## Results

The male sample included 14 582 releases among 12 688 individuals over 83 months for an average 175.7 releases per month (mean [SD] age, 35.0 [10.8] years; 133 Asian and Pacific Islander [0.9%], 4079 Black [28.0%], 4208 Hispanic [28.9%], and 6117 White [41.9%]). The female sample included 3269 releases among 2537 individuals over 58 months, for an average 56.4 releases per month (mean [SD] age, 34.9 [9.8] years; 328 Black [10.0%], 225 Hispanic [6.9%], 2545 White [77.9%]). Characteristics of the released population were relatively consistent over the study period. The most apparent discontinuity was the dramatically higher proportion with serious mental illness in 2019 and 2020 (527 of 2040 releases [25.8%] and 604 of 1878 releases [29.6%], respectively, vs 105 of 2040 releases [5.2%] in 2018 among male individuals; 395 of 495 releases [71.7%] in 2019 vs 35 of 593 releases [5.9%] in 2018 among female individuals), but this coincided with when MADOC broadened the definition (Table 1).

**Table 1.** Characteristics of Individuals Released From Massachusetts Department of Correction by Year^a^

Characteristics	Participants, No. (%)						
	2014^b^	2015	2016	2017	2018	2019	2020^b^
**Male (n = 14 582 releases)**							
Total annual releases	2187	2309	2137	1991	2040	2040	1878
Monthly releases, mean (SD)	182.3 (19.8)	192.4 (28.5)	178.1 (28.3)	165.9 (20.1)	170.0 (26.9)	170.0 (31.6)	170.7 (47.2)
Age, mean (SD), y	37.5 (11.1)	37.9 (11.1)	38.2 (11.3)	38.5 (11.4)	38.7 (11.5)	39.8 (12.0)	40.2 (12.4)
LOI, mean (SD), d	1183 (1221)	1263 (1438)	1279 (1601)	1279 (1469)	1370 (1835)	1395 (1836)	1531 (2239)
Race and ethnicity							
Hispanic (any race)	600 (27.4)	631 (27.3)	596 (27.9)	584 (29.3)	581 (28.5)	609 (29.9)	607 (29.7)
Non-Hispanic Asian and Pacific Islander	23 (1.1)	21 (0.9)	16 (0.8)	18 (0.9)	20 (1.0)	15 (0.7)	20 (1.0)
Non-Hispanic Black	630 (28.8)	637 (27.6)	602 (28.2)	543 (27.3)	586 (28.7)	521 (25.5)	560 (27.4)
Non-Hispanic White	914 (41.8)	966 (41.8)	867 (40.6)	821 (41.2)	832 (40.8)	882 (43.2)	835 (40.9)
Other^c^	S	13 (0.6)	22 (1.0)	25 (1.3)	21 (1.0)	13 (0.6)	21 (1.0)
Unknown	S	41 (1.8)	34 (1.6)	0	0	0	0
Serious mental illness^d^	133 (6.1)	133 (5.8)	134 (6.3)	116 (5.8)	105 (5.2)	527 (25.8)^c^	604 (29.6)^c^
Governing offense^e^							
Property	288 (13.2)	317 (13.7)	256 (12.0)	245 (12.3)	252 (12.4)	239 (11.7)	234 (11.5)
Drug	577 (26.4)	555 (24.0)	470 (22.0)	483 (24.3)	490 (24.0)	485 (23.8)	479 (23.5)
Person^e^	869 (39.7)	953 (41.3)	935 (43.8)	853 (42.8)	859 (42.1)	874 (42.8)	870 (42.6)
Sex^e^	176 (8.1)	189 (8.2)	168 (7.9)	143 (7.2)	165 (8.1)	189 (9.3)	179 (8.8)
Other	277 (12.7)	295 (12.8)	308 (14.4)	267 (13.4)	274 (13.4)	253 (12.4)	281 (13.8)
MATRI program^f^	Se	42 (1.8)	71 (3.3)	137 (6.9)	149 (7.3)	129 (6.3)	0^f^
Released on parole	542 (24.8)	523 (22.7)	344 (16.1)	308 (15.5)	389 (19.1)	420 (20.6)	655 (32.1)
**Female (n = 3269 releases)**							
Total annual releases	NA	797	735	649	593	495	NA
Monthly releases, mean (SD)	NA	66.4 (5.4)	61.3 (7.8)	54.1 (8.8)	49.4 (8.4)	49.5 (10.9)	NA
Age, mean (SD), y	NA	34.5 (9.8)	35.6 (9.9)	35.2 (9.8)	35.9 (9.8)	37.2 (9.8)	NA
LOI, mean (SD), d	NA	219.6 (554.4)	233.5 (538.3)	217.0 (444.8)	230.6 (585.3)	245.3 (677.3)	NA
Race and ethnicity	NA						NA
Hispanic (any race)	NA	51 (6.4)	51 (6.9)	40 (6.2)	37 (6.2)	46 (8.4)	NA
Non-Hispanic Asian and Pacific Islander^g^	NA	S	S	0	S	S	NA
Non-Hispanic Black	NA	89 (11.2)	64 (8.7)	69 (10.6)	58 (9.8)	48 (8.7)	NA
Non-Hispanic White	NA	601 (75.4)	567 (77.1)	495 (76.3)	456 (76.9)	426 (77.3)	NA
Other^c^^,^^g^	NA	S	S	45 (6.9)	S	S	NA
Serious mental illness^d^	NA	69 (8.7)	55 (7.5)	54 (8.3)	35 (5.9)	395 (71.7)^d^	NA
Governing offense^e^	NA						NA
Property	NA	272 (34.1)	239 (32.5)	193 (29.7)	157 (26.5)	154 (28.0)	NA
Drug	NA	161 (20.2)	151 (20.5)	147 (22.7)	121 (20.4)	115 (20.9)	NA
Person^e^	NA	165 (20.7)	163 (22.2)	159 (24.5)	136 (22.9)	123 (22.3)	NA
Sex^e^^,^^g^	NA	12 (1.5)	S	S	S	11 (2.2)	NA
Other^g^	NA	187 (23.5)	S	S	S	S	NA
MATRI program^f^	NA	22 (2.8)	28 (3.8)	57 (8.8)	39 (6.6)	34 (6.2)	NA
Released on parole	NA	120 (15.1)	126 (17.1)	96 (14.8)	107 (18.0)	114 (20.7)	NA

Abbreviations: LOI, length of incarceration; MADOC, Massachusetts Department of Correction; MATRI, Medication Assisted Treatment Reentry Initiative; NA, not applicable; S, suppressed.

^a^ Sample characteristics in this table are aggregated by calendar year of release in order to minimize suppression of small cells; however, the main analysis (interrupted time series using segmented regression) uses data aggregated by calendar month of release.

^b^ For the female sample, the 2014 and 2020 columns are labeled NA because this sample consisted of criminally sentenced women who were released from January 2015 to October 2019; the male sample consisted of criminally sentenced men who were released from January 2014 to November 2020.

^c^ Other includes individuals classified in the MADOC data set as “American Indian or Other [race], non-Hispanic.”

^d^ In 2019, MADOC started using a broader definition of serious mental illness that added more diagnoses such as depression and anxiety disorders, as directed by the Criminal Justice Reform Act passed by the Massachusetts legislature in 2018 (Section 86).^57^

^e^ Governing offense refers to the crime which carries the longest sentence, among whatever crime(s) of which someone has been convicted. “Person” refers to “crimes against the person” that are not sexual in nature and includes assault, battery, robbery, and murder; “sex” refers to “crimes against the person” that are considered sexual in nature, as defined by Massachusetts General Laws chapter 265 (Crimes against the person).^58^ The Massachusetts Department of Correction classifies these 2 categories (person and sex) as violent offenses and classifies the other 3 categories (property, drug, and other) as nonviolent offenses.

^f^ MADOC’s MATRI program (which offered prerelease injectable naltrexone and postrelease linkage to ongoing treatment for eligible individuals with alcohol use disorder and/or opioid use disorder) was first implemented in 2014 and then ended in April 2019 when it was replaced by MADOC’s more comprehensive medications for addiction treatment (MAT) program, which offered naltrexone and buprenorphine (and eventually methadone starting in December 2020).

^g^ Per the privacy protection policy of Massachusetts Department of Public Health, cells containing 1-10 individuals have been suppressed, and, where necessary for polytomous variables, additional cells have been reciprocally suppressed to prevent back-calculation of the primary suppressed cell.

**Figure 1.**
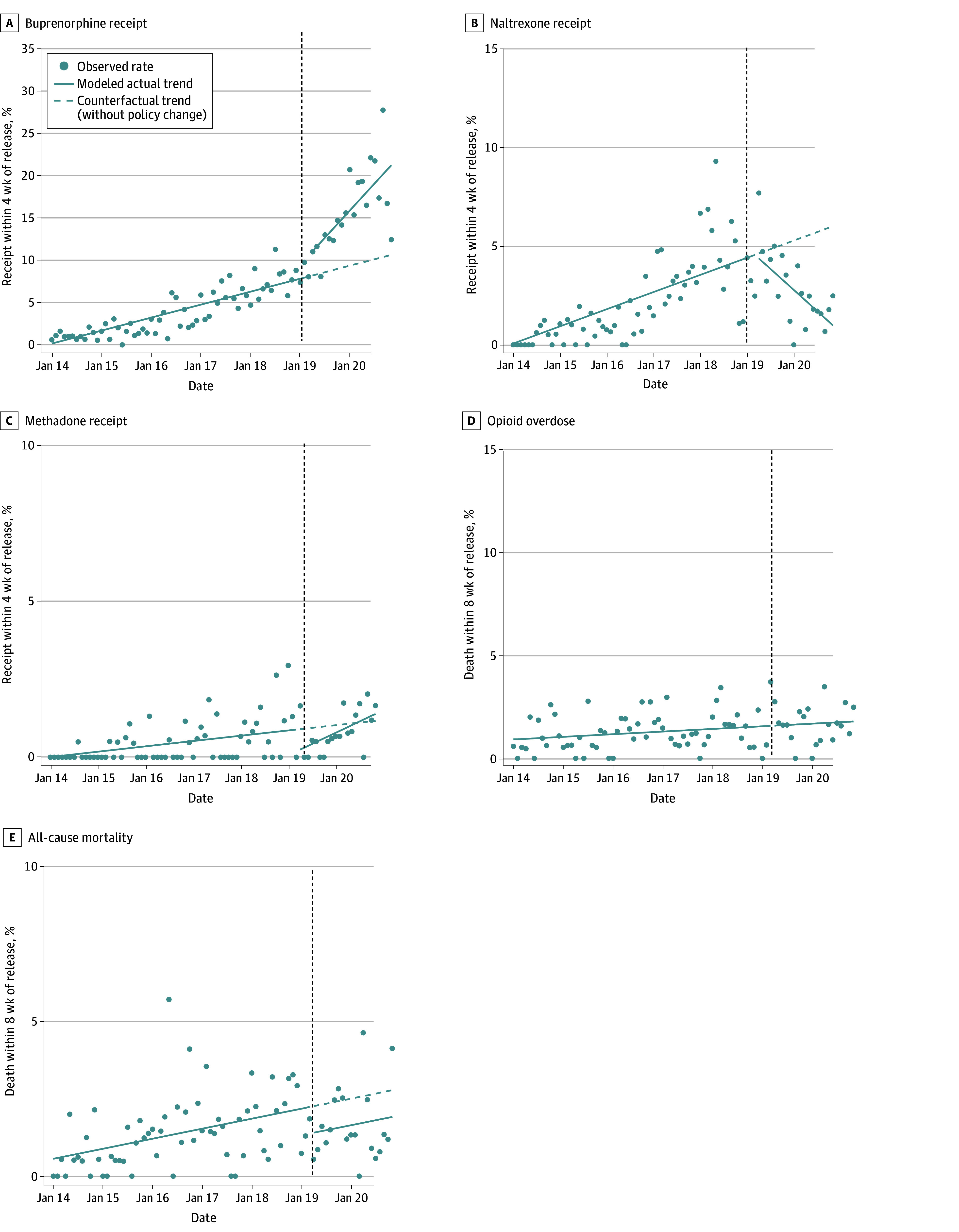
Postrelease Rates of Medication Receipt, Opioid Overdose, and Death for Male Cohort Before and After Buprenorphine Became Available During Incarceration Vertical dotted lines indicate start of intervention (April 2019); scatterplots, observed monthly measures of outcomes; solid lines, actual modeled trends using final segmented linear regression models; dashed lines, counterfactual trends using only the baseline term(s) in the final segmented linear regression model, ie, expected trends without the intervention. In cases where the final model did not include any postintervention change terms, actual and counterfactual postintervention trends are the same.

Among male participants, 988 releases (6.8%) received buprenorphine, 331 (2.3%) received naltrexone, 74 (0.5%) received methadone, 200 (1.4%) had an overdose, and 213 (1.5%) died after release. In the interrupted time series analysis, by November 2020 (20 months postimplementation), the monthly rate of postrelease buprenorphine receipt was higher than would have been expected under baseline trends, 21.2% vs 10.6% of monthly releases (corresponding to an additional 18.6 releases per month on average). Naltrexone receipt was lower than expected, 1.0% vs 6.0% (8.8 fewer releases per month). Methadone receipt was higher but not statistically significantly different than expected, 1.4% vs 1.2%. Opioid overdose was no different than expected, 1.8% (both change terms dropped from final model). All-cause mortality was lower than expected, 1.9% vs 2.8% (1.5 fewer deaths per month) (Table 2 and Figure 1).

**Table 2.** Segmented Linear Regression Models of Changes in Outcome Measure Trends After Intervention

Outcome	Regression model coefficients, % of monthly releases (95% CI)^a^^,^^b^				Cumulative difference in monthly event rate by end of follow-up^b^^,^^c^^,^^d^	
	Baseline level (first month rate estimate)^e^	Baseline trend (preimplementation)	Change in level (postimplementation)	Change in trend (postimplementation)	Percentage points (95% CI)	Total releases, No./mo^f^
**Male**						
Medication receipt ≤4 wks of release						
Buprenorphine	NA	0.13 (0.11 to 0.14)^g^	2.46 (0.46 to 4.45)^g^	0.41 (0.25 to 0.56)^g^	10.6 (8.6 to 12.6)^g^	18.6
Naltrexone	NA	0.07 (0.06 to 0.08)^g^	NA	−0.25 (−0.33 to −0.17)^g^	−5.0 (−6.6 to −3.4)^g^	−8.8
Methadone	NA	0.01 (0.01 to 0.02)^g^	−0.70 (−1.22 to −0.18)^g^	0.05 (0.01 to 0.09)^g^	0.2 (−0.3 to 0.7)	0.4
Health outcomes ≤8 wks of release						
Opioid overdose	0.91 (0.52 to 1.30)^g^	0.01 (0 to 0.02)^g^	NA	NA	NA	NA
Death (any cause)	0.54 (0 to 1.08)	0.03 (0.01 to 0.04)^g^	−0.86 (−1.68 to −0.04)^g^	NA	−0.9 (−1.7 to 0)^g^	−1.5
**Female**						
Medication receipt ≤4 wks of release						
Buprenorphine	2.76 (0.43 to 5.09)^g^	0.12 (0.04 to 0.19)^g^	NA	3.15 (2.29 to 4.01)^g^	22.1 (16.2 to 28.0)^g^	12.4
Naltrexone	1.23 (−0.44 to 2.91)	0.10 (0.05 to 0.16)^g^	NA	−0.54 (−1.16 to 0.08)	−3.7 (−8.0 to 0.5)	−2.1
Methadone	2.09 (1.20 to 2.98)^g^	−0.02 (−0.04 to 0.01)	NA	NA	NA	NA
Health outcomes ≤8 wks of release						
Opioid overdose	4.67 (3.13 to 6.21)^g^	0.04 (−0.01 to 0.09)	−2.00 (−4.65 to 0.65)	NA	−2.0 (−4.6 to 0.6)	−1.1
Death (any cause)	NA	0.03 (0.02 to 0.04)^g^	NA	NA	NA	NA

Abbreviation: NA, not applicable.

^a^ For each outcome, results are shown for the final model selected, after terms with *P* > .20 were removed via stepwise backward elimination.

^b^ For regression model coefficients, NA indicates that a term was dropped from the model during stepwise backward elimination because *P* > .20. For the cumulative change, NA indicates that it was not computed because both of the postintervention change terms (level and trend) were dropped from the final model.

^c^ Cumulative difference is calculated in the final month of follow-up as the difference between the modeled outcome rate (estimated using the final model) and the counterfactual outcome rate (estimated using only the baseline terms in the final model, ie, intercept and/or baseline linear trend depending on which one[s] remained in final model after backward elimination). Therefore, the cumulative difference captures any immediate postintervention change in level as well as the cumulative effects of any postintervention change in trend by the end of follow-up. We report this rate difference in its original units, change in percentage of releases per month, and also convert it to more interpretable units, change in number of releases per month.

^d^ Because the male and female samples had different study period end dates, their respective cumulative rate differences by the end of follow-up are calculated in different months: November 2020 for male sample vs October 2019 for female sample.

^e^ The intercept represents the model estimate of the outcome rate for those in the first study month: January 2014 for the male cohort and January 2015 for the female cohort. Like other regression coefficients, NA for the intercept means that the coefficient had *P* > .20 and was removed from the model via stepwise backward elimination; in such cases, the model-estimated baseline level in the first study month was approximately 0.

^f^ The units of the cumulative difference in event rates were converted by multiplying the percentage point difference by the average number of releases per month across the entire study period.

^g^ *P* ≤ .05.

Monthly releases in the male sample were elevated in 3 early months of the COVID-19 pandemic (April to June, 2020) but returned to usual levels thereafter (eFigure 1 in Supplement 1). In the sensitivity analysis where the male sample follow-up was truncated after December 2019 to avoid the possible influence of the COVID-19 pandemic, results for all 5 outcomes were similar to the main analysis (eTable 3 and eFigure 2 in Supplement 1).

**Figure 2.**
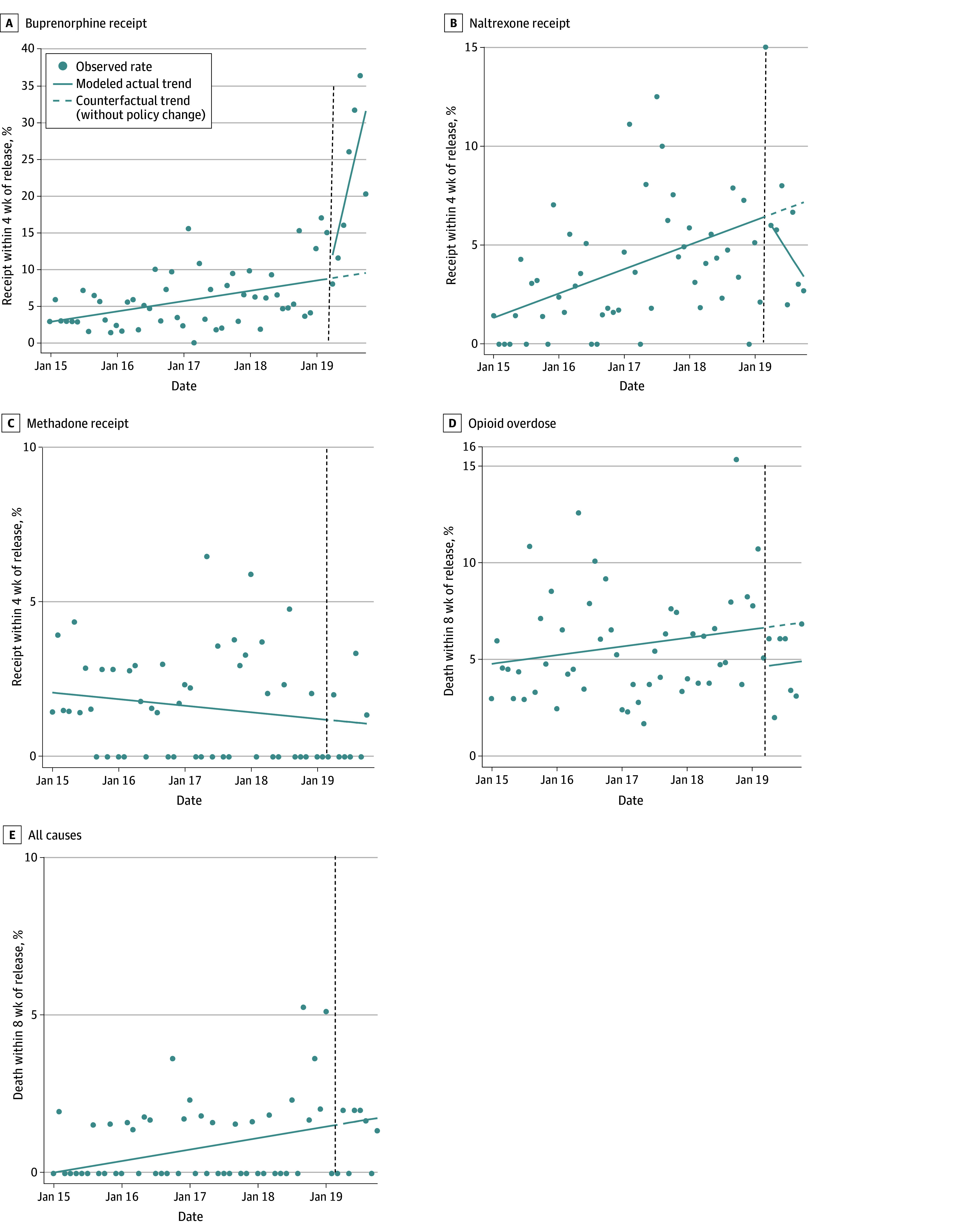
Postrelease Rates of Medication Receipt, Opioid Overdose, and Death for Female Cohort Before and After Buprenorphine Became Available During Incarceration Vertical dotted lines indicate start of intervention (April 2019); scatterplots, observed monthly measures of outcomes; solid lines, actual modeled trends using final segmented linear regression models; dashed lines, counterfactual trends using only the baseline term(s) in the final segmented linear regression model, ie, expected trends without the intervention. In cases where the final model did not include any postintervention change terms, actual and counterfactual postintervention trends are the same.

Among female participants, 239 releases (7.3%) received buprenorphine, 125 (3.8%) received naltrexone, 55 (1.7%) received methadone, 182 (5.6%) had an overdose, and 30 (0.9%) died after release. In the interrupted time series analysis, by October 2019 (7 months postimplementation) the monthly rate of buprenorphine receipt was higher than expected, 31.6% vs 9.5% (corresponding to an additional 12.4 releases per month on average); naltrexone receipt was lower but not statistically significantly different than expected, 3.4% vs 7.2%. Methadone receipt was no different than expected, 1.1% (both change terms dropped from final model). Opioid overdose was lower but not statistically significantly different than expected, 4.8% vs 6.8%. All-cause mortality was no different than expected, 1.6% (both change terms dropped from final model) (Table 2 and Figure 2).

## Discussion

In this interrupted time-series analysis of releases from a state prison system, implementation of buprenorphine treatment during incarceration was associated with higher postincarceration MOUD receipt, largely driven by higher buprenorphine delivery, and lower postincarceration naltrexone receipt. Our real-world findings in this statewide analysis suggest that implementing MOUD treatment during incarceration might improve postrelease medication receipt, which in turn could reduce relapses and overdoses during this high-risk period.

Of note, the rise in buprenorphine coincided with a decline in extended-release naltrexone once patients in MADOC had access to both medications. One plausible explanation is that many patients preferred buprenorphine, which would align with the experience in Rhode Island: after the unified jail and prison system began offering all 3 forms of MOUD in 2016, very few patients (1%) chose naltrexone, while 56% received methadone and 43% buprenorphine.^44^ Little research has examined patients’ MOUD preferences in carceral settings,^45,46^ but qualitative studies have highlighted the importance of having options so that patients can make individualized treatment choices.^47,48^ Indeed, despite a decrease in naltrexone, we found that overall postrelease MOUD receipt increased in the setting of more treatment options during incarceration. Likewise, future research should investigate how postrelease MOUD receipt may have changed after December 2020 when MADOC added methadone to its program—a medication that had very low postincarceration receipt rates throughout our study period.

We suspect that the provision of MOUD while incarcerated drove the substantial increase in postrelease buprenorphine receipt. Our data source lacked information on treatment received in prison, but a published MADOC report^49^ with data from October through December 2019 (a few months after implementation) showed that a mean of 76.7 male patients and 53.3 female patients each month received buprenorphine in the pilot facilities—a treatment volume large enough to translate into the observed postrelease treatment increases. Nevertheless, additional mechanisms (ie, spillover effects) may have also played important roles. For example, in the male-population prisons that did not offer buprenorphine, MADOC’s buprenorphine prescribers could send a prescription to a community pharmacy so that a patient could start buprenorphine upon release without having to wait for an appointment with a community clinician. Therefore, our study highlights a need for further research to understand more fully how this policy change affected treatment access and patient outcomes.

The respective findings in our male and female samples were qualitatively concordant with one another. The most striking difference between the samples was that postincarceration buprenorphine receipt rose more sharply among female participants after the intervention, which could be attributed to multiple different factors. Because both of MADOC’s female-population prisons implemented buprenorphine in April 2019, the entire female population gained access from the start, compared with only 2 out of the 11 male-population MADOC prisons. Additionally, the female sample in MADOC may have had a higher prevalence of OUD and therein a greater proportion who stood to gain from access to buprenorphine, which was evidenced by their higher postrelease overdose rates (in this study and others^50,51^) and would mirror prevalence estimates in national carceral samples.^52,53,54,55^ Furthermore, the female MADOC sample includes some county jail populations, which typically have a higher prevalence of substance use disorders than prison populations.^53^

Although our study may have been underpowered for these rarer outcomes, we nonetheless found a small decrease in postrelease mortality in the male cohort (statistically significant) and a small decrease in postrelease overdose in the female cohort (although not statistically significant). These findings add to a small collection of studies assessing population-level outcomes associated with implementing a MOUD program in a statewide carceral system. Preliminary findings from Rhode Island suggested that overdose deaths declined among recently released individuals in the year following the implementation of their carceral MOUD program.^56^

### Limitations

This study had several limitations. First, our findings could have been confounded by secular trends or coinciding events. The same 2018 Massachusetts law, Chapter 208, also mandated that emergency departments have the capacity to initiate buprenorphine treatment, but this requirement did not have a specific deadline for implementation and thus would be an unlikely explanation for the abrupt changes we observed starting in April 2019. Also our sensitivity analysis in the male sample indicated that the COVID-19 pandemic, which emerged midway through postintervention follow-up, did not substantially affect the results. Second, we analyzed all releases without limiting to those with OUD, ie, those who would be eligible for MOUD, although we have no reason to think that there were any substantial shifts in the prevalence of OUD in our sample. Third, our regression analysis did not adjust for potential confounders, but our results did not show any concerning discontinuities (Table 1). Fourth, we also lacked information about MOUD receipt during incarceration, so we could not identify whether an individual’s postincarceration MOUD receipt constituted continuation vs initiation of treatment. Fifth, we could only measure overdoses that led to contact with an ambulance or emergency department, which represent only a fraction of all overdoses. Finally, these study findings from Massachusetts might not generalize to other places.

## Conclusions

Using a multisector, statewide data source, we found that offering buprenorphine treatment during incarceration in a state prison system was associated with substantial increases in buprenorphine and overall MOUD receipt after release, despite a corresponding decline in naltrexone receipt. These findings highlight the importance of providing opioid agonist MOUD to incarcerated patients, because this might improve engagement in life-saving treatment during the high-risk period of postincarceration reentry.

## Notes

Supplement 1. **eTable 1.** Detailed Definitions of Receipt of Medications for Opioid Use Disorder: Databases and Codes Used **eTable 2.** Detailed Definition of Opioid Overdose: Databases and Codes Used **eTable 3.** Sensitivity Analysis: Segmented Linear Regression Results After Excluding the Pandemic Period from Male Sample **eFigure 1.** Total Male Releases Per Month, 2014-2020 **eFigure 2.** Sensitivity Analysis: Segmented Linear Regression Results After Excluding the Pandemic Period from Male Sample

Supplement 2.
Data Sharing Statement
