# Supplementary material for: Prison Buprenorphine Implementation and Postrelease Opioid Use Disorder Outcomes
Source: JAMA Netw Open. 2024 Mar 18;7(3):e242732. doi: 10.1001/jamanetworkopen.2024.2732 (PMC10949092; doi:10.1001/jamanetworkopen.2024.2732)
Supplement: Supplement 1. — eTable 1. Detailed Definitions of Receipt of Medications for Opioid Use Disorder: Databases and Codes Used eTable 2. Detailed Definition of Opioid Overdose: Databases and Codes Used eTable 3. Sensitivity Analysis: Segmented Linear Regression Results After Excluding the Pandemic Period from Male Sample eFigure 1. Total Male Releases Per Month, 2014-2020 eFigure 2. Sensitivity Analysis: Segmented Linear Regression Results After Excluding the Pandemic Period from Male Sample [file jamanetwopen-e242732-s001.pdf]

Supplemental Online Content

Bovell-Ammon BJ, Yan S, Dunn D, et al. Prison buprenorphine implementation and postrelease opioid use disorder outcomes. *JAMA Netw Open.* 2024;7(3):e242732. doi:10.1001/jamanetworkopen.2024.2732

eTable 1. Detailed Definitions of Receipt of Medications for Opioid Use Disorder: Databases and Codes Used

eTable 2. Detailed Definition of Opioid Overdose: Databases and Codes Used

eTable 3. Sensitivity Analysis: Segmented Linear Regression Results After Excluding the Pandemic Period from Male Sample

eFigure 1. Total Male Releases Per Month, 2014-2020

eFigure 2. Sensitivity Analysis: Segmented Linear Regression Results After Excluding the Pandemic Period from Male Sample

This supplemental material has been provided by the authors to give readers additional information about their work.

© 2024 Bovell-Ammon BJ et al. *JAMA Network Open.*

eTable 1. Detailed Definitions of Receipt of Medications for Opioid Use Disorder: Databases and Codes Used

Medication	Database(s)	Specific Codes / Definitions
**Buprenorphine**	All-Payer Claims Database (APCD) – medical claims	HCPCS: J0592, G2068, G2069, G2070, G2071, G2072, G2079, J0570, J0571, J0572, J0573, J0574, J0575
	APCD – pharmacy claims<br><br>[same list of buprenorphine NDCs was used in both APCD and PMP]	We queried RxNorm[a] for NDCs associated with the following Semantic Clinical Drug Form (SCDF) codes, which refer to buprenorphine products used for the treatment of opioid use disorder (including sublingual/buccal forms of buprenorphine and buprenorphine-naloxone, and long-acting injectable/implantable forms of buprenorphine):<br>1542389, 1655031, 1716056, 1729358, 1797649, 1806212, 1996183, 2269593, 371161, 378762<br><br>NDC: 00054017613, 00054017713, 00054018813, 00054018913, 00093537801, 00093537856, 00093537901, 00093537956, 00093572056, 00093572156, 00228315303, 00228315309, 00228315373, 00228315403, 00228315409, 00228315473, 00228315503, 00228315509, 00228315567, 00228315573, 00228315603, 00228315609, 00228315673, 00378092305, 00378092377, 00378092393, 00378092405, 00378092493, 00378876516, 00378876593, 00378876616, 00378876693, 00378876716, 00378876793, 00378876816, 00378876893, 00406192303, 00406192309, 00406192403, 00406192409, 00406800503, 00406802003, 00490005100, 00490005130, 00490005160, 00490005190, 00490705101, 00490705102, 00490705103, 00490705104, 00490705105, 00781721606, 00781721664, 00781722706, 00781722764, 00781723806, 00781723864, 00781724906, 00781724964, 00904700906, 00904701006, 00904715404, 00904715504, 12496010001, 12496010002, 12496010005, 12496030001, 12496030002, 12496030005, 12496120201, 12496120203, 12496120401, 12496120403, 12496120801, 12496120803, 12496121201, 12496121203, 12496127802, 12496128300, 12496128302, 12496130600, 12496130602, 12496131002, 16590066671, 16590066771, 16729054910, 16729055010, 24236030602, 35356000430, 35356055530, 35356055630, 42291017430, 42291017490, 42291017530, 42858050103, 42858050203, 42858060103, 42858060203, 43063066706, 43063075306, 43598057901, 43598057930, 43598058001, 43598058030, 43598058101, 43598058130, 43598058201, 43598058230, 47781035503, 47781035511, 47781035603, 47781035611, 47781035703, 47781035711, 47781035803, 47781035811, 47781071203, 47781071211, 49349042102, 49349055402, 49999039515, 49999039530, 49999063830, 49999063930, 50090157100, 50090292400, 50090580500, 50268014411, 50268014415, 50268014511, 50268014515, 50268014550, 50383028733, 50383028793, 50383029433, 50383029493, 50383092493, 50383093093, 51129376201, 51129376202, 51862060830, 52125064902, 52125067802, 52427069203, 52427069211, 52427069403, 52427069411, 52427069803, 52427069811, 52427071203, 52427071211, 52440010014, 52959030430, 52959074930, 53217013830, 53217024630, 53217032801, 54123011430, 54123090730, 54123091430, 54123092930, 54123095730, 54123098630, 54569573901, 54569573902, 54569639900, 54569640800, 54569657800, 54868570700, 54868570701, 54868570702, 54868570703, 54868570704, 54868575000,
	Prescription Monitoring Program (PMP)<br><br>[same list of buprenorphine NDCs was used in both APCD and PMP]	

© 2024 Bovell-Ammon BJ et al. *JAMA Network Open.*

		55154496200, 55154496204, 55154496206, 55700014730, 55700018430, 55700030230, 55700030330, 55700090130, 55887031204, 55887031215, 55887031230, 55887031290, 58016098800, 58016098801, 58016098802, 58016098803, 58016098804, 58016098805, 58016098806, 58016098807, 58016098808, 58016098809, 58016098810, 58016098812, 58016098814, 58016098815, 58016098816, 58016098818, 58016098820, 58016098821, 58016098824, 58016098825, 58016098826, 58016098827, 58016098828, 58016098830, 58016098832, 58016098835, 58016098836, 58016098840, 58016098842, 58016098844, 58016098845, 58016098848, 58016098850, 58016098856, 58016098860, 58016098867, 58016098869, 58016098870, 58016098871, 58016098872, 58016098873, 58016098875, 58016098876, 58016098877, 58016098879, 58016098880, 58016098881, 58016098882, 58016098883, 58016098884, 58016098887, 58016098889, 58016098890, 58016098891, 58016098892, 58016098893, 58016098896, 58016098897, 58016098898, 58016098899, 58118017608, 58118017708, 58118050108, 58118050208, 58118315608, 58284010014, 59385001201, 59385001230, 59385001401, 59385001430, 59385001601, 59385001630, 60429058611, 60429058630, 60429058633, 60429058711, 60429058730, 60429058733, 60505705300, 60505705305, 60505705400, 60505705405, 60687048111, 60687048121, 60687049211, 60687049221, 60687062611, 60687062665, 60687063711, 60687063765, 60846097003, 60846097103, 61786067802, 61786091102, 61786091202, 62175045232, 62175045832, 62756045964, 62756045983, 62756046064, 62756046083, 62756096964, 62756096983, 62756097064, 62756097083, 63629403401, 63629403402, 63629403403, 63629409201, 63629409202, 63629507401, 63629712501, 63629712502, 63629712503, 63629712504, 63629712505, 63629712506, 63629712507, 63629712508, 63629712601, 63629712602, 63629712603, 63629712604, 63629712605, 63629712606, 63629712607, 63629712608, 63629712609, 63629726901, 63629727001, 63629727002, 63629947501, 63629948201, 63629948301, 63874108403, 63874108503, 63874117303, 64725093003, 64725093004, 64725192403, 64725192404, 65162041503, 65162041509, 65162041603, 65162041609, 67046099030, 67046099130, 67046099230, 67046099330, 67046099430, 67046099530, 67046099630, 67046099730, 67046099830, 67046099930, 67544048132, 68071068160, 68071259203, 68071291503, 68258299103, 68308020230, 68308020830, 69189059101, 70518044200, 70518065200, 70518065201, 70518065202, 70518071100, 70518071101, 70518071102, 70518100700, 70518155700, 70518162500, 70518168400, 70518201400, 70518201401, 70518221600, 70518221700, 70518221800, 70518222600, 70518222601, 70518222602, 70518222603, 70518231100, 70518232700, 70518312900, 70518338900, 70518348700, 70518360300, 71335035301, 71335035302, 71335035303, 71335035304, 71335035305, 71335035306, 71335035307, 71335035308, 71335095001, 71335095002, 71335095003, 71335095004, 71335095005, 71335095006, 71335095007, 71335095008, 71335115400, 71335115401, 71335115402, 71335115403, 71335115404, 71335115405, 71335115406, 71335115407, 71335115408, 71335115409, 71335116300, 71335116301, 71335116302, 71335116303, 71335116304, 71335116305, 71335116306, 71335116307, 71335116308, 71335116309, 71335129601, 71335129602, 71335137800, 71335137801, 71335137802, 71335137803, 71335137804, 71335137805, 71335137806, 71335137807, 71335137808, 71335137809, 71335151401, 71335151402, 71335165301,

© 2024 Bovell-Ammon BJ et al. *JAMA Network Open.*

		71335172001, 71335172002, 71335172500, 71335172501, 71335172502, 71335172503, 71335172504, 71335172505, 71335172506, 71335172507, 71335172508, 71335172509, 71335185800, 71335185801, 71335185802, 71335185803, 71335185804, 71335185805, 71335185806, 71335185807, 71335185808, 71335185809, 76420053430, 76519117000, 76519117001, 76519117002, 76519117003, 76519117004, 76519117005
**Naltrexone**	APCD – pharmacy claims	We queried RxNorm[a] for NDCs of injectable extended-release naltrexone used for the treatment of opioid use disorder.<br><br>NDC: 63459030038, 63459030042, 65757030001, 65757030003, 65757030038, 65757030042, 65757030101, 65757030202, 65757030302, 65757036000
	APCD – medical claims	HCPCS: J2315, G2073<br>ICD-10-PCS: HZ94ZZZ
**Methadone**	APCD – medical claims	HCPCS: H0020, G2067, G2078, S0109<br>ICD-10-PCS procedure: HZ91ZZZ, HZ81ZZZ
	Bureau of Substance Addiction Services (BSAS) – treatment enrollment records	Record of enrolment in "methadone treatment", "outpatient-based opioid treatment (OBOT)", or "Project MAT" (i.e. medication for addiction treatment) program; and where there is NO corresponding record for Naltrexone or Buprenorphine receipt in APCD or PMP, as above.

Abbreviations: APCD, All-Payer Claims Database; PMP, Prescription Monitoring Program; HCPCS, Healthcare Common Procedure Coding System; ICD-10-PCS, International Classification of Diseases, Tenth Revision, Procedure Coding System; NDC, National Drug Code; BSAS, Bureau of Substance Addiction Services.
[a] RxNorm (https://www.nlm.nih.gov/research/umls/rxnorm/overview.html), the normalized naming system and data library for generic and branded drugs produced by the US National Library of Medicine (NLM). Using the NLM's online interface, RxMix (https://mor.nlm.nih.gov/RxMix/), we queried the RxNorm library for NDCs of buprenorphine and naltrexone products used for the treatment of opioid use disorder.

© 2024 Bovell-Ammon BJ et al. *JAMA Network Open.*

eTable 2. Detailed Definition of Opioid Overdose: Databases and Codes Used[a]

Construct	Database(s)	Details & Codes
Hospital or emergency department encounter for acute opioid overdose[b]	Acute Care Hospital Case Mix file:<br>• Emergency Department visits<br>• Inpatient Hospital encounters<br>• Hospital Outpatient Observation encounters	ICD-9-CM: 965.00, 965.01, 965.02, 965.09, 970.1, E850.0, E850.1, E850.2<br>ICD-10-CM: T40.0X1A, T40.0X2A, T40.0X3A, T40.0X4A, T40.0X1D, T40.0X2D, T40.0X3D, T40.0X4D, T40.1X1A, T40.1X2A, T40.1X3A, T40.1X4A, T40.1X1D, T40.1X2D, T40.1X3D, T40.1X4D, T40.2X1A, T40.2X2A, T40.2X3A, T40.2X4A, T40.2X1D, T40.2X2D, T40.2X3D, T40.2X4D, T40.3X1A, T40.3X2A, T40.3X3A, T40.3X4A, T40.3X1D, T40.3X2D, T40.3X3D, T40.3X4D, T40.4X1A, T40.4X2A, T40.4X3A, T40.4X4A, T40.4X1D, T40.4X2D, T40.4X3D, T40.4X4D, T40.601A, T40.601D, T40.602A, T40.602D, T40.603A, T40.603D, T40.604A, T40.604D, T40.691A, T40.692A, T40.693A, T40.694A, T40.691D, T40.692D, T40.693D, T40.694D
Ambulance encounter for acute opioid overdose[b]	Massachusetts Ambulance Trip Record Information System (MATRIS)	Massachusetts Department of Public Health developed and validated a standardized algorithm which assesses whether an ambulance encounter record was related to an acute opioid overdose (see validation study by Jones and colleagues [2023][c]). The version used in our study was described on pages 240-253 in the 7th version of PHD's Data Dictionary from March 2023 (technical document titled "PHD 2.0 Analytic Data Dictionaries Part1 v7 3.2023") downloaded from https://www.mass.gov/doc/phd-20-analytic-data-dictionaries-part1-v7-32023/download (last accessed 3/24/2023). Generally, updated technical documentation for the Massachusetts Public Health Data Warehouse is publicly available on the PHD webpage (https://www.mass.gov/info-details/public-health-data-warehouse-phd-technical-documentation).
Opioid-related overdose death[b]	Death Certificates from the Registry of Vital Records and Statistics	The Massachusetts Department of Public Health developed a standardized assessment of death certificates (detailed below) which identifies an opioid-related death based on ICD-10 codes or, if ICD-10 codes were not yet available for a specific record, based on a literal search of text fields on the death certificate. Because final determination of cause(s) of death from the Office of the Chief Medical Examiner often lags the date of death, some death records from 2019 and 2020 had not yet been updated with final ICD-10 codes by the time of this study. (Note: By Massachusetts law, the Office of the Chief Medical Examiner is notified of all deaths related to a drug poisoning and reviews many of them.)<br><br>If ICD-10 codes available, then an opioid-related death was identified if:<br>BOTH<br>• Underlying Cause of Death field included any of the following ICD-10 codes for poisonings/overdoses: X40-X44, X60-X64, X85, or Y10-Y14;<br>AND<br>• Contributing Cause of Death fields included any of the following ICD-10 codes for opioid poisoning: T40.0, T40.1, T40.2, T40.3, T40.4, and T40.6.

© 2024 Bovell-Ammon BJ et al. *JAMA Network Open.*

		**OR**<br><br><u>If ICD-10 codes not available, then an opioid-related death was identified based on a literal search of text fields for any of the following 45 opioid-related terms</u>:<br>• OPIOID, ACTIQ, DOLOPHINE, MPHINE, DEMEROL, LEVPHANOL, LTAB, CODEINE, PENTAZOCINE, HEROIN, VICODIN, OXYCODONE, ENDOCET, DARVON, SUBLIMAZE, ULTRAM, PERCOCET, HYDROCODONE, OXYMPHONE, HYDROMPHONE, OPIUM, METHADONE, OPIATE, FENTANYL, PROPOXYPHENE, ANPP, 47700, PIPERIDINE, BUPRENPHINE, SUBOXONE, SUBUTEX, TRAMADOL, FENTANIL, DARVOCET, DEXTRPHAN, DILAUDID, EDDP, MEPERIDINE, OXYCONTIN, TAPENTADOL, HERION, OPIOD, OPOID, W-18, W18

Abbreviations: PHD, Public Health Data Warehouse; ICD-10, International Classification of Diseases, Tenth Revision; ICD-9-CM, International Classification of Diseases, Ninth Revision, Clinical Modification; ICD-10-CM, ICD-10 Clinical Modification.

[a] This approach to identifying opioid-related overdoses in the Public Health Data Warehouse was developed by the Massachusetts Department of Public Health. For convenience, we provide this table to the constructs, datasets, codes, and definitions used in this approach. The details of the statistical code used to generate overdose variables are provided in the PHD technical documentation, which is generally available to the public on the PHD webpage (https://www.mass.gov/info-details/public-health-data-warehouse-phd-technical-documentation). The most recent version used in our study was described on pages 293-301 in the 7th version of PHD's Data Dictionary, published March 2023 (technical document titled "PHD 2.0 Analytic Data Dictionaries Part1 v7 3.2023") downloaded from https://www.mass.gov/doc/phd-20-analytic-data-dictionaries-part1-v7-32023/download (last accessed 3/24/2023).

[b] If an individual with an ambulance encounter for an opioid overdose also had a hospital or emergency department (ED) encounter for an overdose on the same day or on the following day, then these two were combined and considered to be the same opioid overdose event. Similarly, if someone with an ambulance or hospital/ED encounter for an opioid overdose also had an opioid-related death on the same day or within the following 3 days, then these were all considered to be the same opioid overdose event (a fatal opioid overdose).

[c] Reference for validation study: Jones K, Bernson D, Fillo KT, Bettano AL. Redefining and categorizing emergency medical service opioid-related incidents in Massachusetts. *Addiction.* Published online February 11, 2023:add.16148. doi:10.1111/add.16148

© 2024 Bovell-Ammon BJ et al. *JAMA Network Open.*

eTable 3. Sensitivity Analysis: Segmented Linear Regression Results After Excluding the Pandemic Period from Male Sample[a]

	Sensitivity Analysis Regression Model Coefficients[b]				Cumulative Difference in Outcome Rate by December 2019[d] (9 months post-intervention)	
	Baseline Level (Intercept)[c]	Baseline Trend	Change in Level	Change in Trend		
**Male**					Sensitivity Analysis Models[e]	Main Analysis Models[f]
**Medication receipt within 28 days after release, %[g]**						
Buprenorphine	NA	0.13*** (0.12, 0.14)	NA	0.68*** (0.46, 0.91)	+6.15* (4.17, 8.13)	+6.11* (4.82, 7.40)
Naltrexone	-0.57 (-1.31, 0.16)	0.08*** (0.06, 0.10)	2.57* (0.65, 4.49)	-0.72*** (-1.04, -0.41)	-3.95* (-5.92, -1.97)	-2.26* (-2.97, -1.54)
Methadone	NA	0.01*** (0.01, 0.02)	NA	-0.10* (-0.17, -0.02)	-0.87* (-1.52, -0.22)	-0.29 (-0.60, +0.03)
**Health outcomes within 56 days after release, %[g]**						
Opioid overdose	0.86*** (0.45, 1.28)	0.01* (0.00, 0.02)	NA	NA	NA	NA
Death (any cause)	0.53* (0.01, 1.05)	0.03* (0.01, 0.04)	-0.76 (-1.65, +0.13)	NA	-0.48 (-1.83, +0.87)	-0.86* (-1.67, -0.05)

Abbreviations: NA, not applicable.
Note: For regression model coefficients, NA indicates that a term was dropped from the model during stepwise backward elimination because p>0.20. For the Cumulative Change, NA indicates that it was not computed because both of the post-intervention change terms (level and trend) were dropped from the final model.
* p ≤ .05
*** p < .0001
[a] In this sensitivity analysis, the study period for the male sample was from January 2014 to December 2019. Compared to our main analysis, the end of the study period was truncated so that the COVID-19 pandemic would not distort our estimation of the changes attributable to the intervention of interest, i.e. the implementation of buprenorphine treatment during incarceration in the Massachusetts Department of Correction in April 2019.

[b] For each outcome, results are shown for the final model selected, after terms with p>0.20 were removed via stepwise backward elimination.

[c] The intercept represents the model estimate of the outcome rate for those in the first study month: January 2014 for males and January 2015 for females. Like other regression coefficients, NA for the intercept means that the coefficient had p>0.20 and was removed from the model via stepwise backward elimination; in such cases, the model assumed that the baseline level in the first study month was 0.

[d] Cumulative change was calculated as the difference between the modeled outcome rate (estimated using the final model) and the counterfactual outcome rate (estimated using only the baseline term[s] from the final model, i.e. intercept and/or baseline linear trend depending on which one[s] remained in final model after backward elimination). Therefore, the cumulative change captures any immediate post-intervention change in level as well as the accumulation of any post-intervention trend/slope changes.

[e] Cumulative change by December 2019, calculated from sensitivity analysis models that were fit to male sample data from January 2014 to December 2019.

[f] Cumulative change by December 2019, calculated from main analysis models that were fit to male sample data from January 2014 to December 2020. We included this column so that cumulative change measures in the sensitivity analysis could be compared to results from main analysis.

[g] As in the main analysis (Table 2 in main article), all measures in this table are reported as the percentage (%) of monthly releases that experienced the outcome.

© 2024 Bovell-Ammon BJ et al. *JAMA Network Open.*

eFigure1. Total Male Releases Per Month, 2014-2020

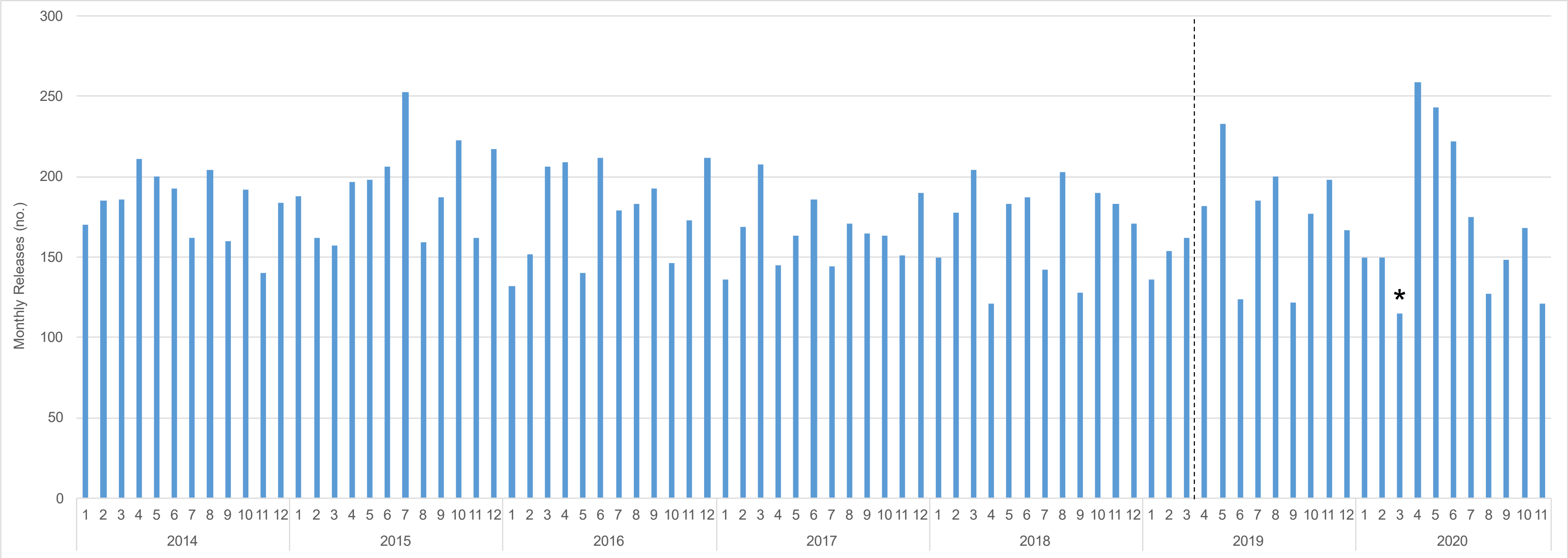

Legend: Vertical dashed bar represents the start of the intervention, i.e. when Massachusetts Department of Correction started offering buprenorphine treatment during incarceration, which began in April 2019. The asterisk (*) marks March 10th, 2020, when the governor of Massachusetts declared a state of emergency due to the coronavirus disease 2019 (COVID-19) pandemic. For context, the Massachusetts Supreme Judicial Court issued a ruling on April 3rd, 2020 (Committee for Public Counsel Services v. Chief Justice of the Trial Court, SJC-12926), that sought to reduce population density in county jails and state prisons, but the ruling primarily pertained to individuals in pre-trial detention and allowed for most of these individuals to be released on personal recognizance. The court stated that it did not have authority to order early release of sentenced individuals and merely urged jurisdictions to expedite the existing parole processes as much as possible. Because our study only includes the sentenced population, the onset of the pandemic had a noticeable but limited impact on monthly releases in 2020.

© 2024 Bovell-Ammon BJ et al. *JAMA Network Open.*

eFigure 2. Sensitivity Analysis: Segmented Linear Regression Results After Excluding the Pandemic Period from Male Sample

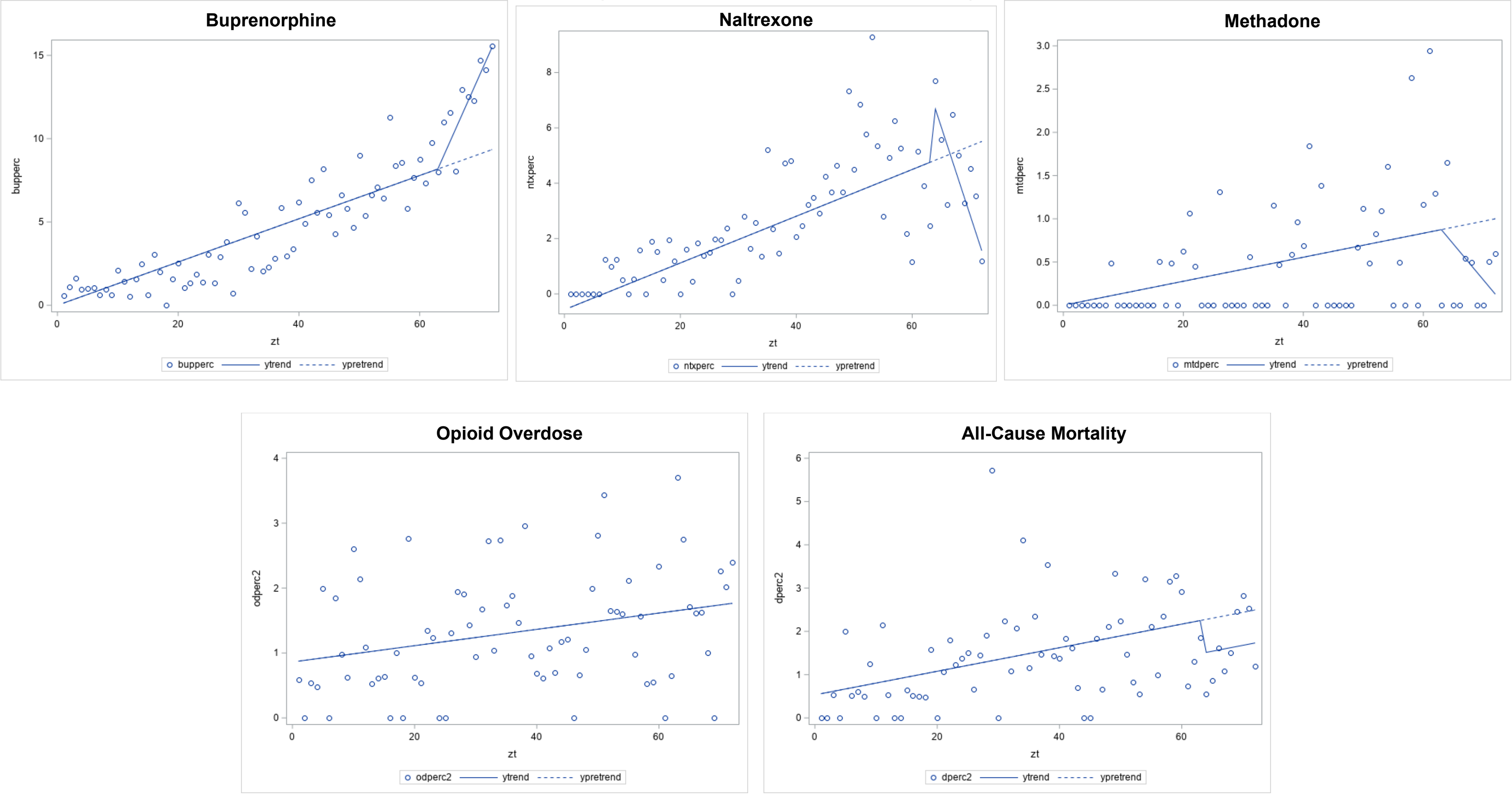

Legend: Scatterplots and model trend lines (including counterfactual post-intervention trend line) are shown for all five post-release outcomes (in order from left to right, top to bottom): buprenorphine receipt, naltrexone receipt, methadone receipt, opioid-related overdose, and death from any cause.

© 2024 Bovell-Ammon BJ et al. *JAMA Network Open.*
