# Supplementary material for: Prison Buprenorphine Implementation and Postrelease Opioid Use Disorder Outcomes
Source: JAMA Netw Open. 2024 Mar 18;7(3):e242732. doi: 10.1001/jamanetworkopen.2024.2732 (PMC10949092; doi:10.1001/jamanetworkopen.2024.2732)
Supplement: Supplement 2. — Data Sharing Statement [file jamanetwopen-e242732-s002.pdf]

Data Sharing Statement

Bovell-Ammon. Prison Buprenorphine Implementation and Postrelease Opioid Use Disorder Outcomes. *JAMA Netw Open*. Published March 18, 2024.
doi:10.1001/jamanetworkopen.2024.2732

Data
Data available: No

Additional Information
Explanation for why data not available: The statistical/analytic code used in this study is available from the authors upon reasonable request. However, the data used in this study cannot be shared by the authors because the data source, the Massachusetts Public Health Data Warehouse (PHD), is restricted and managed by the Massachusetts Department of Public Health. Requests for access to this data source should be directed to this agency (DPH.PHD@mass.gov). General information about PHD, including technical documentation, is available at: https://www.mass.gov/public-health-data-warehouse-phd
